# Effects of Nanoemulsions’ Droplet Size and Natural Antioxidants’ Hydrophilicity on Oxidative Stability and Mechanical Properties of Alginate Beads Filled with Linseed Oil Nanoemulsion

**DOI:** 10.3390/foods15030515

**Published:** 2026-02-02

**Authors:** Zahra Rahiminezhad, Sara Esteghlal, Mohammad Hadian, Gholam Reza Mesbahi, Mohammad-Taghi Golmakani, Seyed Mohammad Hashem Hosseini

**Affiliations:** 1 Department of Food Science and Technology, School of Agriculture, Shiraz University, Shiraz 84334-71946, Iran; 2 Department of Food Science and Technology, College of Agriculture, Fasa University, Fasa 74616-86131, Iran

**Keywords:** clove essential oil, droplet size, hydrogel bead, linseed oil, oxidation, rosemary extract

## Abstract

This study focused on fabricating linseed oil-in-water nanoemulsions (LON) at different pressures of 50 and 150 bar (named as LON50 and LON150, respectively) using a high-pressure homogenizer. Subsequently, these nanoemulsions were encapsulated in alginate hydrogel beads. It was observed that higher homogenizing pressure led to smaller droplet size (108.57 nm), harder beads (222.54 N), less LON release from the beads, and higher oxidation rate, as well as more reduction in α-linolenic acid content during the storage time. To increase the oxidative stability of LON_150_, natural antioxidants including clove essential oil (CEO), rosemary extract (RE), and a mixture of both (CEO+RE) were separately incorporated into the oil phase of LON (LON_150-CEO_), alginate aqueous dispersion (LON_150-RE_), and both lipid and aqueous phases (named as LON_150-CEO+RE_), respectively. It was shown that LON_150-CEO+RE_ had weaker mechanical properties than LON_150-RE_ and LON_150-CEO_. In addition, this sample (LON_150-CEO+RE_) showed the lowest oxidation rate and the minimum α-linolenic acid loss (9.82%) during storage. The highest LON release rate from the beads was related to LON_150-RE_. The results of this study might help in designing bioactive lipids-filled hydrogel beads with appropriate chemical stability and mechanical properties.

## 1. Introduction

Alpha linolenic acid (ALA) as well as other omega-3 (ω-3) fatty acids have different health-promoting effects on the human body such as decreasing the risk of some types of cancers and cardiovascular diseases, improving brain function (especially in early infancy), reducing the risk of cognitive decline, and protection against hormonal and neurological disorders [1,2]. Most seafoods are rich source of omega 3 fatty acids including docosahexaenoic acid (DHA), eicosapentaenoic acid (EPA), and ALA. Linseed oil (LO) is a vegetative alternative source of ω-3. Although ALA makes 55–60% of the total fatty acids of LO, its oral consumption is very limited due to its high oxidation susceptibility [2].

Nowadays, in common Western diets, a sufficient amount of ω-3 fatty acids is not supplied and ω-3/ω-6 is higher than the recommended amount [1,3]. Fortification of food with ω-3 is a way to increase their intake. However, because of the high oxidative susceptibility of omega-3 polyunsaturated fatty acids (PUFAs), very low water solubility, and variable bioavailability, supplementation of food with them is challenging. Oxidation causes a decrease in nutritional value and can produce toxic compounds and off flavors. Application of delivery systems to encapsulate oils rich in ω-3 (PUFA) is a strategy to overcome these limitations. Emulsion, nanoemulsion, and filled hydrogel as various kinds of emulsion-based delivery systems are appropriate choices to encapsulate ω-3-rich oils since they can incorporate the hydrophobic phase into aqueous phase-based foods [1]. To overcome the instability of conventional emulsions against environmental stresses, Fioramonti et al. [2] utilized a multilayer oil-in-water (O/W) emulsion for the encapsulation of LO. In another study, LO was emulsified using surfactant, casein, and whey protein, and then the impact of each one on the oxidative stability and coalescence during in vitro digestion was investigated [4]. While emulsions and nanoemulsions are commonly employed for PUFA encapsulation, their effectiveness in preventing oxidation is limited. The thin layer of emulsifier surrounding the droplets fails to fully shield the fatty acid chains near the interface from exposure to oxygen and pro-oxidants. Moreover, the high interfacial area, especially in nanoemulsions and high light penetration, promotes oxidation in these systems. Therefore, additional steps are necessary to be taken to improve the oxidative stability of emulsions encapsulating PUFA. Hydrogels are three-dimensional (3D) networks of hydrophilic (bio) polymers that have attractive characteristics, making them an appropriate candidate in various fields such as delivery systems, wound healing, and tissue engineering. They can absorb and retain considerable amounts of water, swell without dissolving, and keep their structure in the presence of cross-linkers [5]. Hydrogels can be found in different physical forms such as filled hydrogel beads which can improve bioavailability, dispersibility, and chemical stability of encapsulated lipophilic compounds [1,6]. Food-grade hydrogels are usually fabricated from protein and/or polysaccharide through different methods such as coacervation, injection, thermodynamic incompatibility, templating, molding, and anti-solvent precipitation [7]. Protein-polysaccharide hydrogels are increasingly regarded as ‘smart materials’ for biotechnology due to their tunable responsiveness to environmental stimuli such as pH, temperature, and ionic strength, which dictates their release and mechanical behaviors [8]. Alginate beads are widely used to encapsulate hydrophobic materials because of their low cost, biocompatibility, biodegradability, and ability to easily form a 3D network in the vicinity of Ca^2+^ and other multivalent ions [6,9]. Alginate as a linear hydrocolloid mainly contains 1,4-linked β-D-mannuronic acid (M) and α-L-guluronic acid (G) residues. There can be electrostatic cross-linking between the anionic –COO^−^ groups of the poly-L-guluronate chains and Ca^2+^ (or other multivalent ions), forming an egg-box structure [10]. Extrusion is the most widely used technique to prepare (nano)emulsion-filled alginate hydrogel beads, in which (nano)emulsion and alginate dispersion are mixed and dripped into a gelling solution (such as CaCl_2_) [1,11]. Kour and co-workers [12] successfully used ionotropic hybrid biopolymeric hydrogel beads composed of alginate, chitosan, gelatin, and polyethylene oxide to encapsulate curcumin-loaded nanoemulsions. They report that complexation between curcumin and calcium ions in the hydrogel beads affected the release kinetic of curcumin. Elsewedy et al. encapsulated tea tree oil nanoemulsion into a hydrogel system and indicated that it was an efficient system for topical delivery of neomycin [13]. Mostaghimi et al. indicated that loading thyme and clove essential oils into alginate beads had a protective effect on the essential oils and controlled their release [14]. Chen et al. [1] showed that an alginate hydrogel bead was an effective delivery system to control oxidation of flaxseed oil nanoemulsion. Zhang et al. [6] used lipid-loaded alginate beads and lipid-loaded carrageenan beads for curcumin encapsulation. They reported that the robust structure of the alginate beads could retain the curcumin and lipid droplets through the gastrointestinal tract (GIT), while the carrageenan beads had a fragile structure and released curcumin and lipid droplets through the GIT. Emulsion-filled alginate beads were used by Feng et al. [10] to encapsulate resveratrol and α-tocopherol. They indicated that the concentrations of whey protein isolate (as emulsifier) and oil were important parameters for protection of resveratrol and α-tocopherol, respectively. In another study, encapsulation of beta carotene was carried out within free oil droplets and oil droplet-filled hydrogel beads. The results of this study indicated much higher oxidative stability and lower bioaccessibility in a lipid-loaded filled-hydrogel system compared to beta carotene in unloaded lipid droplets [6]. The effect of alginate, calcium, and tween 60 concentrations on the release of nanoemulsion containing curcumin from alginate beads was investigated by Zeeb et al. [15]. The results showed that higher concentrations of alginate and calcium decreased the nanoemulsion release, while higher concentrations of tween 60 led to an increase in curcumin release.

The addition of antioxidants is another strategy to enhance the oxidative stability of PUFA. Consumers are increasingly seeking natural antioxidants due to issues associated with the consumption of synthetic antioxidants, including health risks (e.g., liver expansion resulting from butylated hydroxytoluene consumption) and the unpleasant taste and odor after degradation [16]. The effectiveness of natural extracts may improve due to the interactions among their different phenolic components. Synergy can occur through various mechanisms. For instance, secondary compounds like rosmarinic acid or eugenol can help regenerate primary antioxidants such as tocopherols or carnosic acid. They can also target different stages of lipid oxidation at the same time [17]. This combined effect means that a whole extract may offer more protection than the individual components alone. This provides a strong reason to use extracts instead of isolated, purified compounds [18]. Antioxidant activity of rosemary extract (RE) [19,20,21], clove essential oil (CEO) [15,22,23], and cinnamon essential oil [24] has been proved by different researchers. This antioxidant capacity was related to presence of phenolic compounds in RE and CEO [16].

While there are several reports on the effect of adding natural antioxidants and oil droplet size on oxidative stability of O/W emulsions [25,26,27], the impact of these factors on the chemical stability of nanoemulsions loaded in hydrogel structures is still unexplored. This study uniquely investigates how homogenizer pressure (induced droplet size variations) along with hydrophilicity of natural antioxidants affect LO oxidation after immobilization of LO-in-water nanoemulsion in alginate hydrogel beads, a topic that has been scarcely explored.

## 2. Materials and Methods

### 2.1. Materials

Linseed oil (LO) was obtained by cold pressing of *Linum usitatissimim* seeds in a local market in Shiraz, Iran. In order to prevent interfering with the results, no antioxidant was added to the LO. All of the needed amounts of linseed oil were obtained from the same batch. Rosemary and clove were bought from a local market in Shiraz and then authenticated at School of Agriculture, Shiraz University (Shiraz, Fars, Iran). CEO was extracted by hydro-distillation method as described by [22], and freeze-dried aqueous extract of rosemary leaves was obtained based on the method of Gómez-Estaca et al. [28] at Food Science and Technology Department of Shiraz University (Shiraz, Fars, Iran). CEO and RE were stored at −20 °C until use. Tween 80, thiobarbituric acid (TBA), trichloroacetic acid (TCA), ethanol 96%, hydrochloric acid, acetyl chloride, methanol, tert-butylhydroquinone (TBHQ), ethylendiaminetetraacetic acid (EDTA), and 2,2-diphenyl-1-picrylhydrazyl (DPPH) were purchased from Merck Co. (Darmstadt, Germany). Sodium alginate salt, butyl hydroxy toluene, CaCl_2_, and hexane were prepared from analytical grade sources.

### 2.2. Preparation of Nanoemulsions

The nanoemulsions were prepared based on our previous work with some modifications [29]. First, the tween 80 (5% *w*/*w*), as the emulsifier, was mixed with double-distilled water (DDW) and stirred (700 rpm, 15 min). Then, LO was gradually added to distilled water–tween 80 mixture under continuous stirring (700 rpm, 15 min) to reach 10% (*w*/*w*) concentration. In the next step, an Ultra-Turrax T-25-Basci homogenizer (IKA-Werke GmbH + Co. KG, Staufen, Germany) was applied to fabricate the initial coarse emulsion at 12,000 rpm for 5 min. The final nanoemulsions were fabricated by homogenizing the coarse emulsion at two different pressures of 50 bar (LON_50_) and 150 bar (LON_150_) by a high-pressure homogenizer (FBF, Parma, Italy) for seven cycles [30].

### 2.3. Droplet Size and Zeta Potential Measurement

Dynamic light scattering (DLS, SZ-100, Horiba, Kyoto, Japan) was applied to determine the intensity-weighted average droplet size and polydispersity index (PI) of the prepared nanoemulsions. For this purpose, 4 mL of each DDW-diluted sample (in a 1:10 *v*/*v* ratio) was poured into the DLS plastic cell. The zeta potential of the nanoemulsion droplets was determined by DLS in a disposable plastic cell equipped with carbon-coated electrodes (Horiba, Japan). Smoluchowski approximation was used to convert the electrophoretic mobility to zeta potential. Measurements were performed at 25 °C in triplicate, and the data is reported as mean ± SD.

### 2.4. Fabrication of Filled Hydrogel

Based on our previous work [29], the optimum concentrations of sodium alginate and CaCl_2_ solutions to prepare alginate hydrogel beads were 2% and 0.5%, respectively. After stirring overnight at 700 rpm, dispersion of sodium alginate (2%) was mixed with the prepared nanoemulsions (LON_50_ and LON_150_) at a 1:1 mixing ratio. Each mixture was added gradually to calcium chloride solution (0.5%) to form beads. Then, stirring continued for half an hour to complete the hardening process of beads. Afterwards, the beads were separated from the solution and kept at 4 °C [15]. Unloaded LONs were used as control samples.

### 2.5. Release of Nanoemulsions from Filled Hydrogels into DDW

For determining the release of LONs from the beads, a certain number of beads (equal to 2 g) were poured into 15 mL of DDW and stirred at 200 rpm for 120 min. Finally, the absorbance of DDW surrounding the beads was measured at 600 nm at ambient temperature [10]. The value was measured in triplicate.

### 2.6. Mechanical Characteristics

Hardness, chewiness, and cohesiveness of filled beads were measured according to the technique of [10]. Briefly, a Petri dish was filled with beads and analyzed by a texturometer (TA-XTplus, Stable Micro Systems, Surrey, UK). A 35 mm probe at a compression rate of 0.025 mm/s was used for this purpose. The experiment was performed in triplicate, and the values are reported as average ± SD.

### 2.7. Oxidative Stability Measurement

Oxidative stability of unloaded LONs and loaded LONs (in alginate beads) was measured every 7 days during 8 weeks of storage at 4 °C. Prior to analysis at each interval, the LON needed to be extracted from the alginate hydrogels. Briefly, 4 g of the filled beads was added to 20 mL of saturated EDTA solution to weaken the alginate cross-links by calcium binding. The mixture remained constant for 24 h, and then a microfuge (MIKRO120, Hettich, Kirchlengern, Germany) was used to apply shear force (12,000 rpm, 10 min). The creamy part in the upper phase which contained LON was collected. To measure thiobarbituric acid-reactive substances (TBARS), a mixture of TCA (75 g), TBA (1.86 g), HCl (8.8 mL, 12 M), and DDW (414 g) was prepared, and 97 mL of it was admixed with 3 mL of ethanolic solution of BHT (2%). An amount of 2 mL of this solution was mixed with 2 mL of the obtained creamy supernatant and heated in a water bath (95 °C, 15 min). Then, centrifuging (SW14R, Froilabo, Meyzieu, France) of the mixture was performed at 10,000× *g* for 10 min followed by cooling to 25 °C. The upper phase was collected, and its absorbance was determined at 532 nm. Standard curve prepared by 1,1,3,3-tetraethoxypropane was used to measure the TBARS amount [31]. All results are expressed as mean standard error of three experiments.

### 2.8. Gas Chromatography Analysis of LO

Fatty acids profile of linseed oil in free and encapsulated forms was determined during the storage time (8 weeks, 4 °C). LON was extracted from the beads as explained in Section 2.7. Next, to break the LON and extract the oil phase, each sample was boiled in a water bath for 15 min. Then, to extract the oil phase, the heated LON was mixed with 3 mL of hexane and mixed well for 5 min and then centrifuged at 10,000× *g* for 15 min. The upper phase (LO) was collected, and 500 mg of it was added to 10 mL of methanol/acetyl chloride (1:20 ratio), heated at 85 °C in an oven for 60 min, and then rapidly cooled to ambient temperature. The methyl acetylation process was completed by adding 5 mL of DDW and then mixing for 5 min. In the next step, 2 mL of hexane containing TBHQ (0.01%) was mixed and centrifuged at 4000 rpm for 5 min at room temperature. An amount of 1 μL of the supernatant was injected into the gas chromatograph (B420A, BEIFEN, Beijing, China) equipped with a BPX70 column which had a film thickness of 0.25 µm, inner diameter of 0.25 mm, and length of 60 m. Nitrogen gas and Polymer Biscyanopropylsiloxane-silphenylene were chosen as stationary and mobile phases, respectively. A flame-ionized type detector was used. The initial temperature was 90 °C and raised 3 °C/min until reaching 210 °C and remained at this temperature for about 25 min. The injector and detector temperatures were 250 °C and 300 °C, respectively. Chromatography Data Handling System software 7.3 was used for analyzing the obtained chromatograms [31].

### 2.9. DPPH Scavenging Assay of CEO and RE

The antioxidant activity of CEO and RE was measured according to scavenging activity of 2,2-diphenyl-1-picrylhydrazyl (DPPH). The DPPH solution was prepared in methanol (0.1 mM) and then mixed with each sample at a 1:1 mixing ratio. The used concentrations of CEO in this test were 10, 50, 100, 500, and 1000 ppm, and that of RE were 100, 1000, 5000, 10,000, and 20,000 ppm. The mixtures of DPPH solution samples were kept in darkness until completing the reaction between them. After 1 h, absorbance (A) of the mixtures was determined at 517 nm. The blank and control samples were methanol and methanolic solution of DPPH, respectively. IC_50_ (concentration of CEO or RE which leads to 50% inhibition) was calculated using the graph of absorbance vs. concentration. The test was performed thrice, and the following equation was used to determine the DPPH scavenging capacity (%):

$$\%DPPH\;scavenging\;capacity=(\frac{A_{control}-A_{sample}}{A_{control}})\times 100$$

### 2.10. Incorporation of Rosemary Extract and Clove Essential Oil

Based on the results of the oxidative stability test, RE and CEO were used to enhance the oxidative stability of alginate beads filled with LON_150_. Three types of hydrogel samples were prepared: (i) loaded LON_CEO_ in which CEO was mixed with the oil phase of LO before preparation of nanoemulsions, (ii) loaded LON_RE_ in which RE was added to sodium alginate dispersion and the resultant hydrogel filled with LON_150_, and (iii) loaded LON_CEO+RE_ which were alginate beads containing RE in the aqueous phase of sodium alginate and filled with LON_150-CEO_. This sample contained both CEO and RE in the oil phase of nanoemulsion and aqueous phase of sodium alginate dispersion, respectively. The concentrations of RE and CEO were chosen based on IC_50_ values.

### 2.11. Properties of Loaded LON_CEO_, LON_RE_, and LON_CEO+RE_

Release of nanoemulsions from loaded LON_CEO_, LON_RE_, and LON_CEO+RE_ and their mechanical properties were based on the method explained in Section 2.5 and Section 2.6, respectively. Stability of LON_CEO_, LON_RE_, and LON_CEO+RE_ against oxidation and their fatty acid profile were determined in a similar manner described in Section 2.7 and Section 2.8, respectively. Unloaded LON_150_ incorporated with CEO was chosen as the control sample.

### 2.12. Statistical Analysis

The tests were performed three times, and the results are shown as mean ± standard deviation. Significant differences between the means were analyzed by analysis of variance using SAS^®^ software (version 9.1, SAS Institute Inc., Cary, NC, USA). Duncan multiple range test was used to compare the means at a confidence level of 0.05.

## 3. Results and Discussion

### 3.1. Particle Size Distribution and Zeta Potential

Particle size and zeta potential of emulsion droplets have key roles in their physical stability. Turbulence and shear force are two pressure-dependent factors that can affect emulsion droplet size [32]. As expected, increasing the homogenizer pressure led to smaller droplet size. Based on Table 1, the LON_50_ and LON_150_ samples showed intensity-weighted average droplet sizes of 222.10 ± 6.91 and 108.57 ± 1.50 nm, respectively. Consistent with these findings, Yuan et al. [32] reported that increasing the homogenizer pressure led to a considerable decrease in nanoemulsion droplet size. The polydispersity index (PI) of LON_50_ and LON_150_ were 0.64 ± 0.04 and 0.38 ± 0.01, respectively, which means that higher homogenization pressure resulted in a more uniform droplet size. The zeta potential values for LON_50_ and LON_150_ (Table 1) were −50.10 ± 2.80 and −41.70 ± 1.68 mV, respectively, indicating good physical stability of the nanoemulsions

### 3.2. DPPH Antioxidant Assay of CEO and RE

The antioxidant activity of clove essential oil (CEO) and rosemary extract (RE) was assessed by measuring the scavenging activity of DPPH°. Both CEO and RE, as reducing agents, can induce a color change in the DPPH° from purple to yellow. The IC_50_, representing the concentration of the test material that causes a 50% reduction in the initial DPPH° absorbance, is reported in Table 2. The IC_50_ values for CEO and RE were found to be 4960 and 290 ppm, respectively. These concentrations (IC_50_) were selected for addition to the oil phase (CEO) and the aqueous alginate phase (RE). This metric (IC_50_) reflects the potency of the antioxidant, with a lower IC_50_ corresponding to a lower concentration needed to achieve 50% radical neutralization, thereby indicating greater efficiency [33].

### 3.3. LON Release from Alginate Hydrogel

#### 3.3.1. Release of LON_50_ and LON_150_ from Alginate Hydrogel Beads

Releasing through alginate hydrogels can occur due to diffusion from the pores of the beads or network degradation [15]. As the beads remained undamaged during this study, the release of encapsulated LONs mainly occurred through diffusion. The porosity in the alginate bead structure depends on alginate concentration, the G-block content, and the number of junction zones.

**Figure 1:**
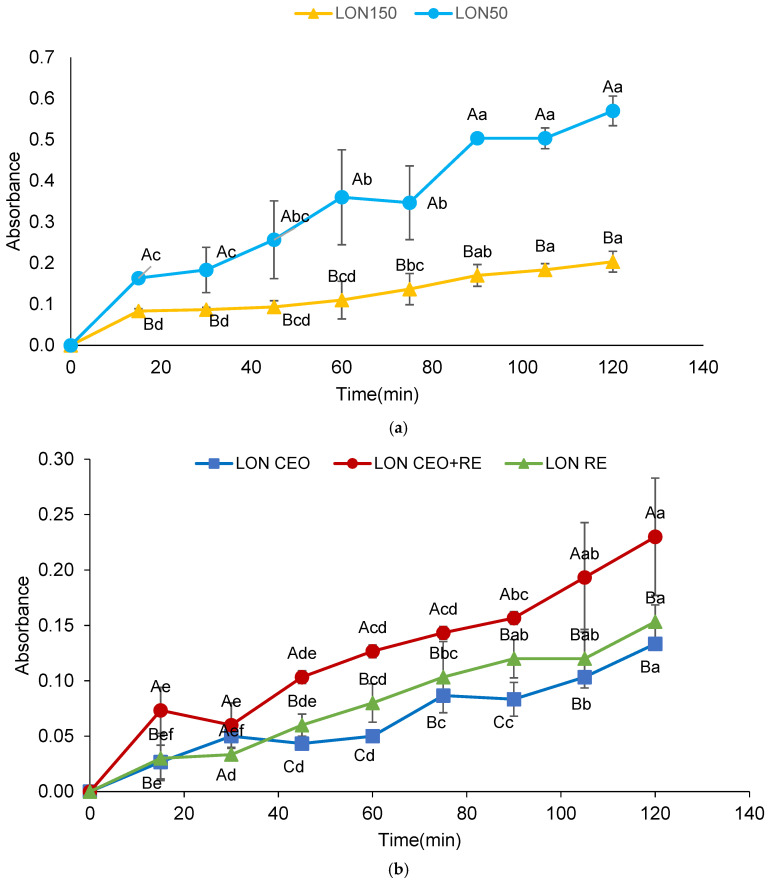
(**a**): Release of linseed oil-in-water nanoemulsions (LONs) prepared at two different homogenizing pressure of 50 (LON_50_) and 150 (LON_150_) bar from alginate hydrogel beads at ambient temperature. (**b**): Release of LONs incorporated with clove essential oil (LON_CEO_), rosemary extract (LON_RE_), and clove essential oil and rosemary extract (LON_CEO+RE_) from alginate hydrogel beads at ambient temperature. At same time, different uppercase letters indicate significant differences (*p* < 0.05). For same samples, different lowercase letters indicate significant differences (*p* < 0.05) over time.

As shown in Figure 1a, both samples exhibited an increasing trend of release during 2 h. The release of LON_50_ (in terms of the amount and the rate) was higher than that of LON_150_, indicating that smaller droplets were released less from the alginate hydrogel. Smaller droplets have a higher specific surface-to-volume ratio and therefore require more surfactant to cover their surface [34]. When the interface is saturated with the small molecules of surfactant, the excess amount of surfactant stays in the continuous phase and forms micelles if the free surfactant concentration exceeds the critical micelle concentration. Small lipophilic compounds can be dissolved in these micelles [35]. Considering that the initial concentration of surfactant was the same in both systems, it is postulated that there was an excess amount of tween 80 present in the LON_50_ (nanoemulsion with larger droplets) system that could form micelles in the aqueous phase and diffuse easily through the pores of the alginate beads. Small portions of the linseed oil might have hydrophobic interactions with the interior of the tween 80 micelles and pass through the pores rapidly, consequently enhancing the linseed oil release in the LON_50_ sample. Another probable reason is that the smaller droplets of LON_150_ were mainly trapped within the water phase of the hydrogel; however, loading of the larger droplets of LON_50_ in the alginate beads was not performed efficiently, and most of the droplets occupied the pores of the hydrogel network, consequently releasing more easily and to a higher extent. Zeeb et al. [15] reported that the fabrication of nanoemulsions by the high-energy method resulted in a higher release of nanoemulsions containing curcumin from sodium alginate beads than nanoemulsions fabricated by the low-energy method.

#### 3.3.2. Release of LON_CEO_, LON_RE_, and LON_CEO+RE_ from Alginate Beads

The release of loaded LON_CEO_, LON_RE_, and LON_CEO+RE_ through the beads is depicted in Figure 1b. In all samples, the release of LON exhibited an increasing trend during the test. Notably, the LON_RE_ sample showed the highest nanoemulsion release. LON_CEO_ and LON_CEO+RE_ had a similar extent of release at the first two points; however, after 30 min, LON_CEO+RE_ exhibited a slightly higher release rate than LON_CEO_. This observation could be attributed to the hydrophilic nature of RE, which could readily diffuse through the alginate beads to DDW surrounding them, leading to an increase in absorbance. The outward diffusion of these hydrophilic compounds may create a subtle convective flow or concentration gradient that enhances the mobility and release of the co-encapsulated nanoemulsion droplets from the bead matrix [36].

### 3.4. Mechanical Properties

#### 3.4.1. Alginate Hydrogel Beads Filled with LON_50_ and LON_150_

Hydrogels, characterized by a cross-linked network of hydrophilic biopolymers with the ability to retain substantial amounts of water, exhibit a wide range of mechanical properties. The results of TPA, including hardness, cohesiveness, and chewiness values, are presented in Table 3. Hardness, representing the required force to compress a sample, was observed to be 222.54 ± 25.65 N for alginate beads containing LON_150_ and 144.09 ± 5.21 N for those containing LON_50_. As explained in Section 3.2, the lower hardness of the hydrogel filled with larger emulsion droplets (LON_50_) can be attributed to the trapping of these droplets within the pores, causing a softening effect on the beads. Additionally, the higher surface-to-volume ratio of small droplets led to increased friction and, consequently, a higher hardness value.

Chewiness, a measure of the required energy and resistance of a solid food during mastication, was found to be 65.61 ± 6.18 N for LON50-filled alginate beads and 102.59 ± 11.14 N for LON150-filled beads. The higher chewiness value of the latter was attributed to its harder structure (higher hardness value).

Cohesiveness, indicating the integrity of a structure due to intermolecular interactions, ranged between 0 and 1. When the encapsulated active ingredient has undesirable taste, keeping the structural integrity of capsules is important to avoid releasing during mastication. The cohesiveness values of LON_50_ (0.72 ± 0.1) and LON_150_ (0.73 ± 0.2)-filled hydrogels were not statistically significant, indicating that both samples remained mostly intact during the double compression test, and cohesiveness was independent from the droplet size of the nanoemulsions. Some research studies have similarly reported the independence of cohesiveness from the concentrations of encapsulated materials, calcium chloride, and alginate [11,29]. However, Lozano-Vazquez et al. [9] reported that the cohesiveness of sodium alginate beads filled with modified tapioca starch varied with different weight ratios of alginate/tapioca starch and alginate/Ca^2+^.

#### 3.4.2. Alginate Hydrogel Beads Filled with LON_CEO_, LON_RE_, and LON_CEO+RE_

In this section, the mechanical properties of alginate beads filled with LON_CEO_, LON_RE_, and LON_CEO+RE_ are discussed (Table 3). The hardness values were 201.61 ± 37.24 N for LON_CEO_, 257.78 ± 86.12 N for LON_RE_, and 124.21 ± 32.59 N for LON_CEO+RE_. Natural antioxidants such as CEO and RE, containing phenolic compounds, can interact with the hydrophilic (such as carboxylic) groups of alginate [37]. These interactions, primarily based on non-polar and van der Waals forces, may explain the increased hardness of beads containing LON_RE_. Chewiness and cohesiveness values were not significantly different (*p* > 0.05) for beads filled with LON_CEO_ and LON_RE_, while those filled with LON_CEO+RE_ showed lower values (52.86 ± 15.69 and 0.68 ± 0.06, respectively). In addition, polyphenols in RE and CEO could chelate Ca^2+^ ions, competing with alginate and potentially weakening the ‘egg-box’ junctions, contributing to the lower hardness of LON_CEO+RE_ beads [10]. Nadavala et al. [37] also explains that concurrent hydrogen bonding between polyphenols and alginate chains may introduce steric effects, altering network density and stiffness.

### 3.5. Oxidative Stability

#### 3.5.1. Free and Loaded LON_50_ and LON_150_

Lipid oxidation susceptibility provides a risk to the quality of various products. The oxidation process in emulsified lipids is intricate and significantly differs from the oxidation of bulk lipids. In oil-in-water emulsions, pro-oxidants such as transition metals and antioxidants coexist in the aqueous phase. Interactions between the oil phase and components in the aqueous phase take place at the interface, initiating oxidation primarily from this location.

**Figure 2:**
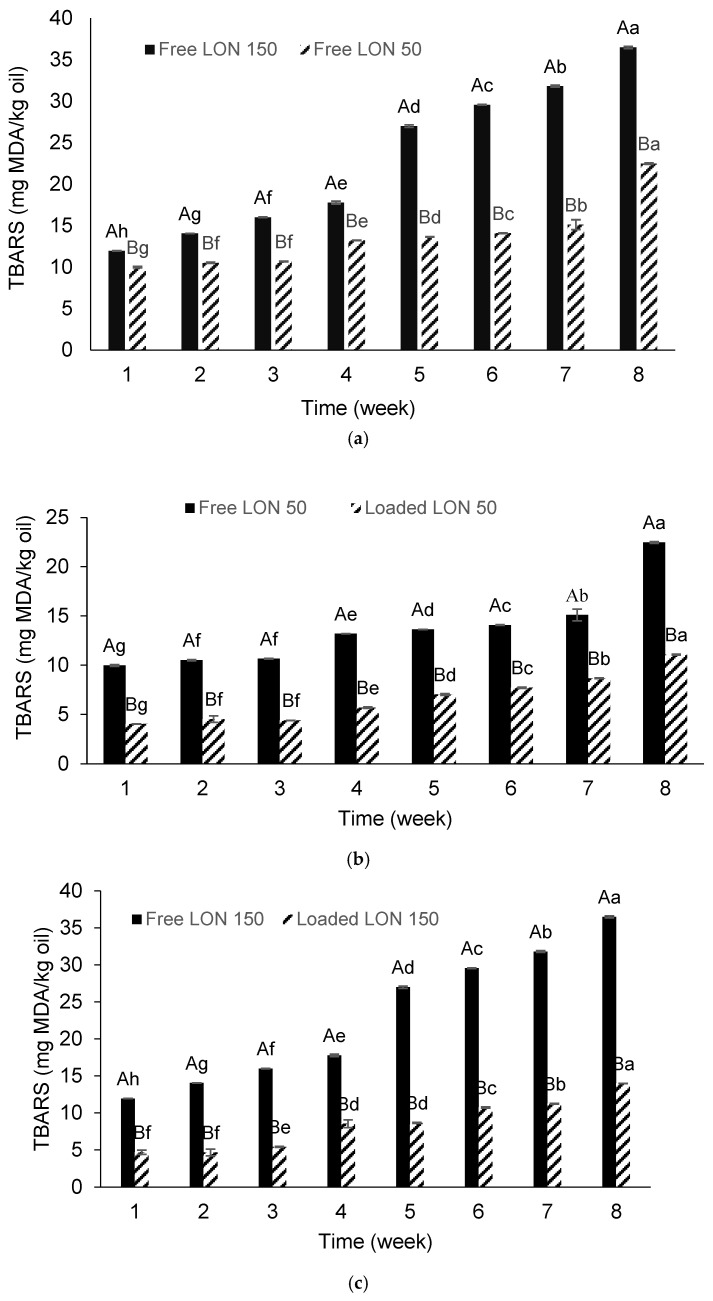

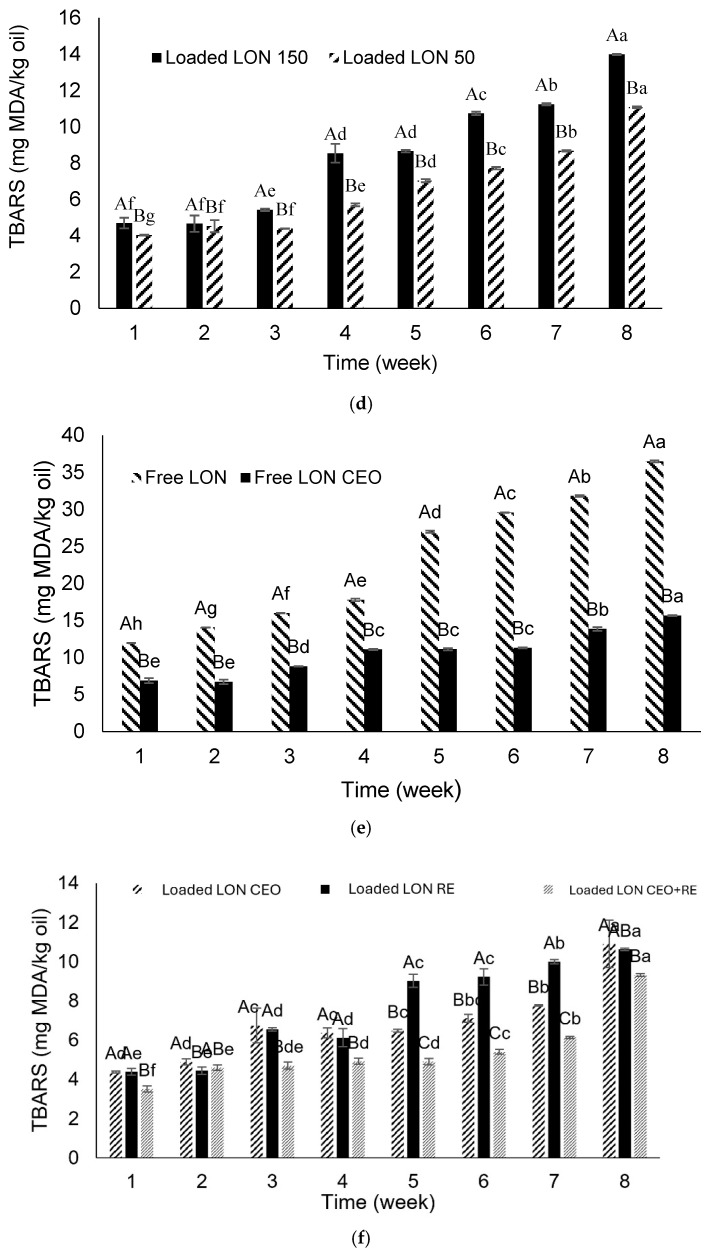
Changes in the thiobarbituric acid-reactive substances (TBARS) of (**a**): free linseed oil-in-water nanoemulsions prepared at homogenization pressure of 50 (LON_50_) and 150 (LON_150_) bar; (**b**): free LON_50_ and loaded LON_50_ in alginate hydrogel beads; (**c**): free LON_150_ and loaded LON_150_; (**d**): loaded LON_50_ and loaded LON_150_; (**e**): free LON_150_ and free LON_150-CEO_ (incorporated with clove essential oil within the oil phase); (**f**): alginate beads loaded with LON_150-CEO_ (containing CEO within the oil phase of nanoemulsion (loaded LON_CEO_), alginate beads containing RE in the aqueous phase of sodium alginate solution which loaded with LON_150_ (loaded LON_RE_), alginate beads containing RE in the aqueous phase of sodium alginate dispersion which loaded with LON_150-CEO_ (loaded LON_CEO+RE_) during storage at 4 °C. At same time of storage, different uppercase letters indicate significant differences (*p* < 0.05). For same sample, different lowercase letters indicate significant differences (*p* < 0.05) over time.

To evaluate the extent of oxidation, TBARS, an index of secondary products of lipid oxidation, was measured during an 8-week storage period at 4 °C for both unloaded (free) and loaded LON_50_ and LON_150_ systems. The choice of this storage temperature aimed to prevent microbial growth in the samples. The results, depicted in Figure 2a, reveal an increase in TBARS levels in both unloaded LON_50_ and LON_150_ systems, with a more pronounced increment in LON_150_, especially from the 4th week onward. The smaller emulsion nanodroplets of LON_150_, due to having a higher specific surface area, provided more opportunities for the interactions between the oil droplets and pro-oxidants as well as dissolved oxygen in the aqueous phase.

Another factor influencing the greater extent of oxidation in LON_150_ is related to the oxidation of the surfactant. Smaller droplets require more surfactants for stabilization, and the hydrophilic head of polysorbate, containing polyethers, is easily oxidized when exposed to transition metals or free radicals, resulting in the production of hydroperoxides and degradation products like TBARS. Additionally, the probable formation of micelles in the LON_50_ sample (emulsion with larger droplets), as explained in Section 3.2, could solubilize lipid hydroperoxides and reduce free radicals in the oil phase, ultimately decreasing TBARS levels. These micelles might also solubilize iron and remove it from the oil droplets, limiting lipid oxidation in oil-in-water emulsions with surfactant micelles in the continuous aqueous phase.

Comparison between loaded and unloaded LONs in Figure 2b,c shows a lower content of TBARS in loaded samples, indicating that alginate beads could protect the emulsified linseed oil from oxidation. Alginate’s ability to chelate metals and to prevent hydroperoxide from decomposition contributed to this protective effect. Similarly, the retardation of oxidation in ω_-3_ oil nanoemulsions loaded in alginate hydrogel, due to the presence of casein as a chelating agent, has been reported [30]. Figure 2d demonstrates that the amount of TBARS in loaded LON_150_ was higher than that in loaded LON_50_, attributing this difference to the reasons mentioned above.

#### 3.5.2. Loaded LON_150-CEO_, LON_150-RE_, and LON_150-CEO+RE_

Considering that LON_150_ (with smaller droplets) exhibited higher oxidative decay, this sample was selected for incorporation with natural antioxidants. Figure 2e, depicting the amount of malondialdehyde in free forms of LON_150-CEO_ and LON_150_ (without any added antioxidant, as a control sample), reveals oxidation in both samples during this study. However, the extent of oxidation in the LON_150-CEO_ sample was significantly lower due to the antioxidant activity of CEO. The antioxidant activity of CEO has been demonstrated in previous studies by [22,23].

A comparison between loaded LON_150-CEO_, LON_150-RE_, and LON_150-CEO+RE_ samples (Figure 2f) shows an increasing trend in TBARS levels in all beads during the storage period of 56 days. The lowest oxidation rate was observed in LON_150-CEO+RE_, attributable to the synergistic effect of CEO and RE. Until the 4th week, there was no significant difference in the amounts of TBARS between LON_150-CEO_ and LON_150-RE_. However, from the 4th week onward, TBARS levels were considerably higher in LON_150-RE_. This difference was attributed to the lower concentration of RE compared to CEO. In this study, natural antioxidants were added based on the measured IC_50_, and RE revealed a lower IC_50_ value (Table 2). The RE was also added to aqueous phase of alginate which might have had a dilution effect as well. The location of the antioxidant is a crucial parameter in its ability to control oxidation. According to the antioxidant polar paradox hypothesis, non-polar antioxidants are more efficient in controlling oxidation in oil-in-water emulsions, as they stay in the oil phase where the oxidation process occurs. Non-polar antioxidants in an oil-in-water emulsion can accumulate at the interface, where the oxidation mechanism initiates. The higher potential of non-polar antioxidants compared to their polar counterparts in retarding oxidation has been reported by other researchers [38,39].

### 3.6. Fatty Acid Profile of Unloaded and Loaded LONs

The GC analysis revealed that the predominant fatty acids in linseed oil were α-linolenic, linoleic, oleic (unsaturated), palmitic, and stearic (saturated) acids. Since polyunsaturated fatty acids are more prone to oxidation, changes in α-linolenic acid (ω-3) and linoleic acid (ω-6) of linseed oil in different samples were analyzed during the storage time at 4 °C. Table 4 shows that the amounts of α-linolenic and linoleic acids decreased in almost all samples during storage, which indicated oxidative degradation. A comparison between antioxidant-free samples demonstrated that the maximum (38.57%) and the minimum (12.55%) decrease in α-linolenic acid content occurred in free LON_150_ (nanoemulsion with smaller droplets) and loaded LON_50_ (alginate beads filled with nanoemulsion with larger droplets), respectively. At the end of storage, the α-linolenic acid content in loaded LONs was higher than that in free LONs, consistent with the TBARS measurements. The results of the fatty acids profile confirmed a higher oxidation rate in nanoemulsions with smaller droplets and the protective effect of alginate hydrogel on linseed oil. This correlation between reduced droplet size and a higher oxidation rate aligns with the established principle that lipid oxidation in emulsions is an interfacial phenomenon, where a larger surface area promotes the reaction between pro-oxidants and unsaturated lipids [40].

The fatty acids profile in free LON_150-CEO_, loaded LON_150-CEO_, loaded LON_150-RE_, and loaded LON_150-CEO+RE_ showed a decreasing trend in α-linolenic and linoleic acids content. However, in these samples the degradation rate was lower than antioxidant-free counterparts, indicating the positive role of natural antioxidants in retarding oxidation. Loaded LON_150-CEO_ and loaded LON_150-CEO+RE_ showed a decrease only in the amount of α-linolenic acid content. Some observed increase in the relative proportion of linoleic acid and saturated fatty acids () during storage is likely an analytical artifact of the GC-FID method. Since the amount of individual fatty acids is determined by normalizing peak areas against the total area, the preferential degradation of chemically less stable fatty acids over time mathematically results in an apparent rise in the relative percentage of other fatty acids.

## 4. Conclusions

In this study, calcium alginate hydrogel beads were filled with linseed oil nanoemulsions (LONs) prepared at two different homogenization pressures, resulting in LONs with different droplet sizes. The results indicated that the oxidative instability and α-linolenic acid degradation were inversely correlated with droplet size (i.e., higher oxidation in nanoemulsions with smaller droplet sizes). Irrespective of droplet size, loading of LONs into alginate hydrogels significantly increased the oxidative stability of nanoemulsions. To mitigate the oxidation rate in LON with smaller droplets, natural antioxidants including rosemary extract (RE), clove essential oil (CEO), and a mixture of them were incorporated into either the lipid phase of the nanoemulsions or the aqueous phase of the alginate dispersion. The findings revealed that the mixture of antioxidants had a better ability to control the oxidation process and prevent the reduction of PUFAs. In addition, observing the mechanical characteristics of different samples demonstrated a better understanding of the texture properties and behavior of the formulated alginate beads for possible further applications. In summary, optimizing droplet size and incorporating a natural antioxidant blend can significantly enhance the oxidative stability of linseed oil within calcium alginate hydrogel beads. This dual strategy effectively preserves α-linolenic acid content, offering a robust framework for stabilizing sensitive bioactive compounds in emulsion-based delivery systems.

## Figures and Tables

**Table 1:** Size and zeta potential of linseed oil-in-water nanoemulsions prepared at homogenizer pressures of 50 (LON_50_) and 150 (LON_150_) bar.

Sample	Size (nm)	PI	Zeta Potential (mV)
LON_50_	222.10 ± 6.91 ^A^	0.64 ± 0.04 ^A^	−50.10 ± 2.80 ^B^
LON_150_	105.57 ± 1.50 ^B^	0.38 ± 0.01 ^B^	−41.70 ± 1.64 ^A^

Data are the average of at least three independent replicates ± standard deviation. In each column, different uppercase letters indicate significant differences (*p* < 0.05).

**Table 2:** IC_50_ values of clove essential oil (CEO) and rosemary extract (RE).

Sample	IC_50_ (ppm)
CEO	4960
RE	290

**Table 3:** Mechanical properties of alginate hydrogel beads filled with linseed oil-in-water nanoemulsions.

Mechanical Properties			
Sample	Hardness (N)	Chewiness (N.mm)	Cohesiveness
Alginate beads filled with LON_50_	144.09 ± 5.21 ^B^	65.61 ± 6.16 ^B^	0.72 ± 0.1 ^A^
Alginate beads filled with LON_150_	222.54 ± 25.68 ^A^	102.59 ± 11.14 ^A^	0.73 ± 0.02 ^A^
Alginate beads filled with LON_CEO_	201.61 ± 37.24 ^B^	97.71 ± 14.22 ^AB^	0.75 ± 0.00 ^A^
Alginate beads filled with LON_RE_	257.78 ± 86.12 ^A^	127.15 ± 50.98 ^A^	0.75 ± 0.01 ^A^
Alginate beads filled with LON_CEO+RE_	124.21 ± 32.59 ^C^	52.86 ± 15.69 ^C^	0.68 ± 0.06 ^B^

Data are the average of at least three independent replicates ± standard deviation. In each column, different uppercase letters indicate significant differences (*p* < 0.05).

**Table 4:** Changes in the relative amount (%) of α-linolenic and linoleic acids present in free and encapsulated linseed oil-in-water nanoemulsions during storage at 4 °C.

Sample	Fatty Acid	Storage Time (Day)						Loss (%)
		10	20	30	40	50	60	
Free LON_50_	C18:2(ω-6)	15.47	15.42	15.01	11.86	11.3	11.15	27.93
	C18:3(ω-3)	55.64	51.26	48.12	42.84	39.85	39.32	29.33
Free LON_150_	C18:2(ω-6)	14.91	16.37	14.86	11.99	10.86	8.96	39.91
	C18:3(ω-3)	53.77	52.41	50.10	41.71	37.06	33.03	38.57
Loaded LON_50_	C18:2(ω-6)	15.62	13.00	15.8	15.11	14.75	13.22	15.36
	C18:3(ω-3)	54.20	53.19	53.88	50.80	52.23	47.04	12.55
Loaded LON_150_	C18:2(ω-6)	18.89	15.65	14.62	15.34	13.26	13.28	29.70
	C18:3(ω-3)	54.89	52.59	53.28	47.31	46.92	46.98	14.41
Free LON_150-CEO_	C18:2(ω-6)	15.16	15.64	14.88	12.03	11.28	10.16	32.98
	C18:3(ω-3)	53.67	52.2	50.15	41.63	39.99	36.15	32.64
Loaded LON_150-CEO_	C18:2(ω-6)	9.35	14.27	15.03	16.57	12.96	14.31	-
	C18:3(ω-3)	61.52	48.62	46.02	47.92	44.62	44.56	27.57
Loaded LON_150-RE_	C18:2(ω-6)	15.05	8.55	11.13	16.57	17.8	12.84	14.68
	C18:3(ω-3)	52.32	42.48	47.07	47.35	44.43	42.37	19.02
Loaded LON_150-CEO+RE_	C18:2(ω-6)	13.35	15.92	16.08	14.19	13.59	13.39	-
	C18:3(ω-3)	53.07	51.57	47.68	48.92	47.61	47.86	9.82

LON_50_ and LON_150_ indicate linseed oil-in-water nanoemulsions prepared at homogenization pressure of 50 and 150 bar, respectively. Loaded LON_150-CEO_, loaded LON_150-RE_, and loaded LON_150-CEO+RE_ indicate alginate beads loaded with LON_150-CEO_ containing CEO within the oil phase of nanoemulsion, alginate beads containing RE in the aqueous phase of sodium alginate solution which loaded with LON_150_, and alginate beads containing RE in the aqueous phase of sodium alginate dispersion which loaded with LON_150-CEO_, respectively.

## Data Availability

The original contributions presented in this study are included in the article. Further inquiries can be directed to the corresponding author.
